# P-2216. The characteristics of human microbiome and fecal resistome in patients with recurrent urinary tract infection

**DOI:** 10.1093/ofid/ofae631.2370

**Published:** 2025-01-29

**Authors:** Bongyoung Kim, Yerin Kim, Yeseul Na, Jeoungyeon Kim, Wooyoung Jang, Ji Won Go, Jinnam Kim, Se Yoon Park, Yangsoon Lee, Hyunjoo Pai, Mina Rho

**Affiliations:** Department of Internal Medicine, Hanyang University College of Medicine, Seongdong-gu, Seoul-t'ukpyolsi, Republic of Korea; Hanyang University Cpllege of Engineering, Seoul, Seoul-t'ukpyolsi, Republic of Korea; Hanyang University Seoul Hospital, Seoul, Seoul-t'ukpyolsi, Republic of Korea; Hanyang University Seoul Hosptial, Seoul, Seoul-t'ukpyolsi, Republic of Korea; Hanyang University College of Medicine, Seoul, Seoul-t'ukpyolsi, Republic of Korea; Hanyang University College of Medicine, Seoul, Seoul-t'ukpyolsi, Republic of Korea; Hanyang University Seoul Hosptial, Seoul, Seoul-t'ukpyolsi, Republic of Korea; Division of Infectious Diseases, Department of Internal Medicine, Soonchunhyang University Seoul Hospital, Seoul, Seoul-t'ukpyolsi, Republic of Korea; Department of laboratory medicine, Hanyang University College of Medicine, Seoul, Seoul-t'ukpyolsi, Republic of Korea; Department of Internal Medicine, Hanyang University College of Medicine, Seongdong-gu, Seoul-t'ukpyolsi, Republic of Korea; Hanyang University, Seoul, Seoul-t'ukpyolsi, Republic of Korea

## Abstract

**Background:**

While uropathogenic E. coli (UPEC) is recognized as a primary causative agent of recurrent urinary tract infections (rUTIs), the factors contributing to its colonization remain poorly understood. The study aims to elucidate the characteristics of the human microbiome in patients with rUTI.

Bacterial proportion in genus level: PCoA (distance: Bray-Curtis dissimilarity)

**Figure:**
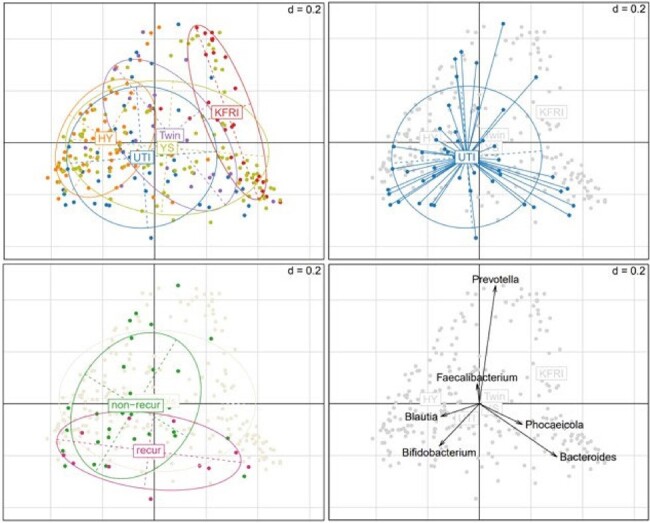

**Methods:**

A prospective study was conducted at a tertiary-care hospital (850 beds) in Korea from August 2021 to November 2023. All female adults (aged ≥19 years) hospitalized with upper UTI were screened daily for inclusion if they met the following criteria: i) *E. coli* was confirmed as causative pathogen, ii) no structural abnormalities in the urinary tract were present, and iii) patients agreed to participate in the study. Individuals diagnosed with UTI after 48 hours of admission or those transferred from other hospitals were excluded. Fresh stool samples were collected after 28 days of completion of antibiotic treatment, and whole metagenome sequences were obtained using Illumina HiSeq sequencing. Patients with a history of UTI within the past year were defined as having rUTI. We analyzed the characteristics of the microbiome in rUTI patients compared with those in non-rUTI patients as well as the healthy population (n=214), which had already been analyzed in other studies.

Bacterial proportion in genus level: Top 7 genera in each dataset

**Figure:**
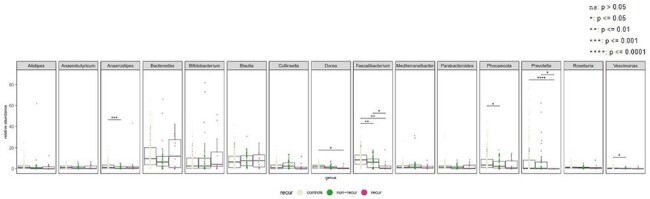

**Results:**

A total of 51 cases were initially enrolled, but three samples failed to pass the quality check. Consequently, 48 cases (including 13 with rUTI) were included in the final analysis. Among UTI patients, the dominant genera observed were Blautia (7.7%), Bacteroides (7.6%), Faecalibacterium (4.3%), and Bifidobacterium (3.5%). Principal coordinates analysis (PCoA) revealed that the overall distribution of the microbiome in rUTI patients exhibited a distinct pattern compared to non-rUTI patients. Additionally, the proportion of Faecalibacterium and Prevotella was lower in rUTI patients compared to non-rUTI patients and the healthy population. Regarding antibiotic resistance genes (ARGs), rUTI patients showed a lower proportion of ACI and CatS compared to non-rUTI patients and the healthy population.

ARG abundance (RPKM) in gene family level: PCoA (distance: Bray-Curtis dissimilarity)

**Figure:**
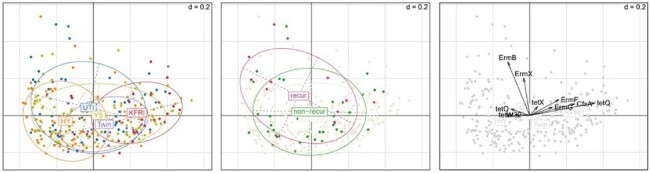

**Conclusion:**

Some differences observed in the distribution of microbiome and resistome between patients with rUTI and non-rUTI or healthy population.

ARG abundance (RPKM) in gene family level

**Figure:**
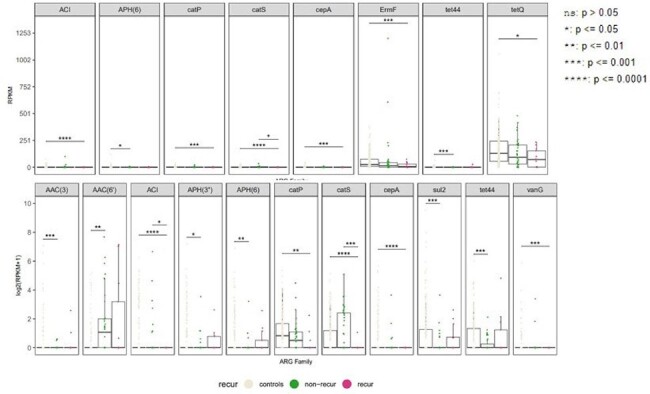

**Disclosures:**

All Authors: No reported disclosures

